# A Pediatric Acute Promyelocytic Leukemia With a Rare Karyotype of ider(17)(q10)t(15;17) and Favorable Outcome: A Case Report

**DOI:** 10.1097/MD.0000000000001778

**Published:** 2015-10-16

**Authors:** Yanli He, Ping Wang, Kaiwei Liang, Xiangjun Chen, Wen Du, Juan Li, Yanjie Hu, Yan Bai, Wei Liu, Xiaoqing Li, Runming Jin, Min Zhang, Jine Zheng

**Affiliations:** From the Center for Stem Cell Research and Application, Institute of Hematology, Union Hospital, Tongji Medical College, Huazhong University of Science and Technology (YH, KL, XC, WD, JL, YH, WL, XL, JZ); Department of Clinical Laboratory, Union Hospital, Tongji Medical College, Huazhong University of Science and Technology (PW); Department of Pediatric, Union Hospital, Tongji Medical College, Huazhong University of Science and Technology (YB, RJ); and Institute of Hematology, Union Hospital, Tongji Medical College, Huazhong University of Science and Technology, Wuhan, China (MZ).

## Abstract

Acute promyelocytic leukemia (APL) is a specific malignant hematological disorder with a diagnostic hallmark of chromosome translocation t(15;17)(q22;q21). As a very rare secondary cytogenetic aberration in pediatric APL, ider(17q) (q10)t(15;17) was suggested to be a poor prognostic factor based on previous case reports.

Here, we report a pediatric APL case with a rare karyotype of ider(17)(q10)t(15;17). Bone marrow aspiration, immunophenotyping, molecular biology, cytogenetic, and fluorescence in situ hybridization (FISH) analyses were performed at initial diagnosis and during the treatment.

A 6-year-old boy was brought to our hospital with the chief complaint of bleeding gums twice and intermittent fever for 3 days in January 2013. He was diagnosed as low-risk APL according to the 2012 NCCN guideline on APL, with the expression of PML-RARA (bcr3 subtype) and the karyotype of 46,XY, der(15)t(15;17)(q22;q21),ider(17)(q10)t(15;17), which was further verified by FISH. The patient was treated through combination all-trans retinoic acid (ATRA) and arsenic with daunorubicin according to the 2012 NCCN guideline for APL. Continuous hematological completed remission (HCR) and major molecular remission (MMR) were achieved with normal karyotype for >28 months after induction chemotherapy.

Different from previously reported cases, this pediatric APL patient with ider(17)(q10)t(15;17) displays favorable clinical outcomes, which might be related to the low-risk classification and arsenic treatment during the treatment. It suggests that ider(17)(q10)t(15;17) may not be the sole determinant for worse outcomes in pediatric APL and implies that more contributed factors should be considered for pediatric APL prognosis.

## INTRODUCTION

The fusion gene PML-RARA generated from chromosome translocation t(15;17)(q22;q21) has been identified as a distinguished marker for acute promyelocytic leukemia (APL).^1^ Classic cytogenetics combined with florescence in situ hybridization (FISH) indicates 90% of typical t(15;17) and some rare case of atypical translocations such as t(5;17), t(11;17), and dup(17)(q21q23) in APL.^2^ In the past decades, owing to the utilization of all-trans-retinoic acid (ATRA) in combination with arsenic and other chemotherapies, APL has been one of most curable leukemia types.^3–5^ As high as 95% of newly diagnosed APL patients with the PML-RARA fusion gene could achieve completed remission (CR) after the treatments, in which ∼80% reaches long-term event-free survival. However, a small population of APL patients still show poor outcomes after post-remission chemotherapy, which may be attributed to some featured genetic abnormalities as well as changes in treatment response, relapse, and clinical pathological characters.^6–9^

The ider(17)(q10)t(15;17)(q22;q12)^6,10–15^ is one of the rare secondary genetic abnormalities in APL. To the best of our knowledge, only 4 pediatric APL cases with ider(17)(q10)t(15;17)(q22;q12) were reported in the literature and all of them died in 1 to 21 months after diagnosis.^16–19^ These data suggest that ider(17)(q10)t(15;17)(q22;q12) is likely to be a poor prognostic marker for pediatric APL. Different from previous reports, we first report a pediatric APL case with the rare karyotype of ider(17)(q10)t(15;17), which still remains CR for 28 months until now.

## MATERIALS AND METHODS

### Case Report

**FIGURE 1:**
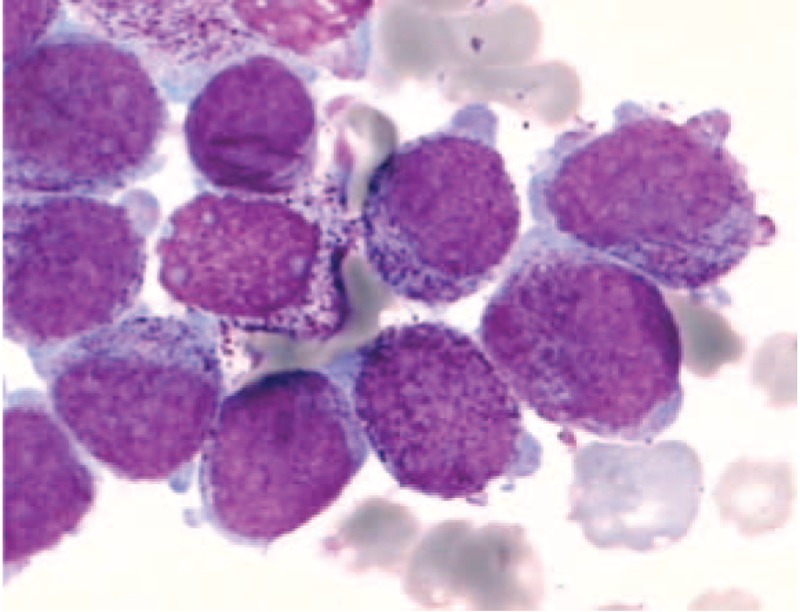
Bone marrow morphology at initial diagnosis. Bone marrow aspiration showed a hypercellular marrow with increased abnormal promyelocytes, which were variable in the cell size and nucleolus, rich in cytoplasm and varying granules, and visible of round or oval, distorted, folded nucleus.

**FIGURE 2:**
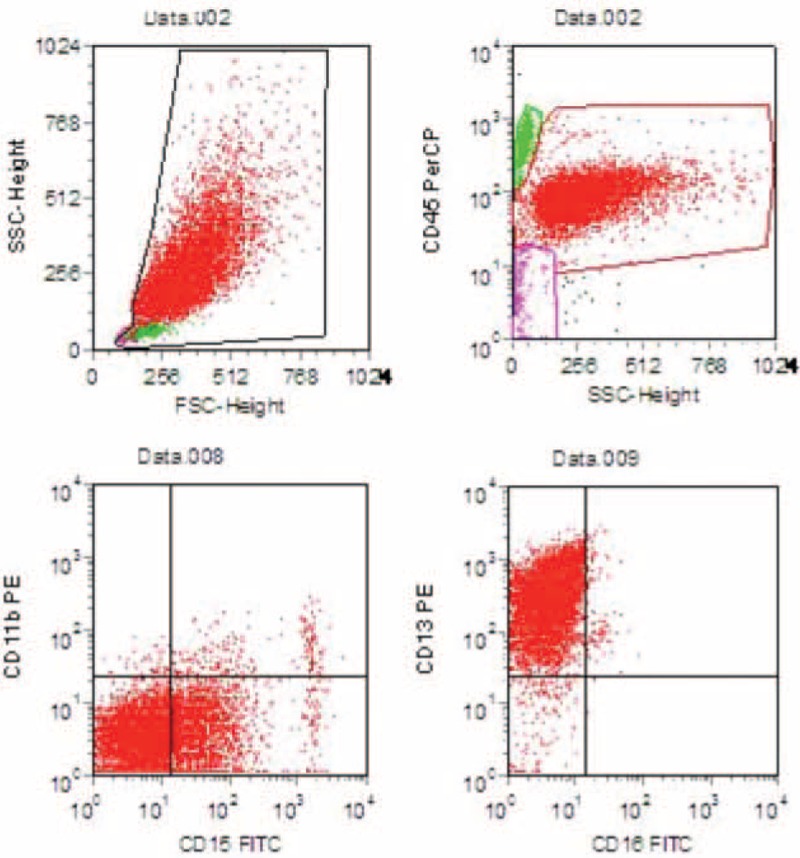
Immunophenotyping of bone marrow at initial diagnosis. In the dot plot of CD45/side scatter (SSC), the abnormal APL leukemia cells were continuously distributed from immature to mature granulocytes cells area. The abnormal promyelocytes comprised 91.5% of the nucleated cells and expressed CD9, CD13, CD15, CD33, CD45, CD64, CD123, Myeloperoxidase (MPO), also with partial expression of CD38 and CD117. APL = acute promyelocytic leukemia.

**FIGURE 3:**
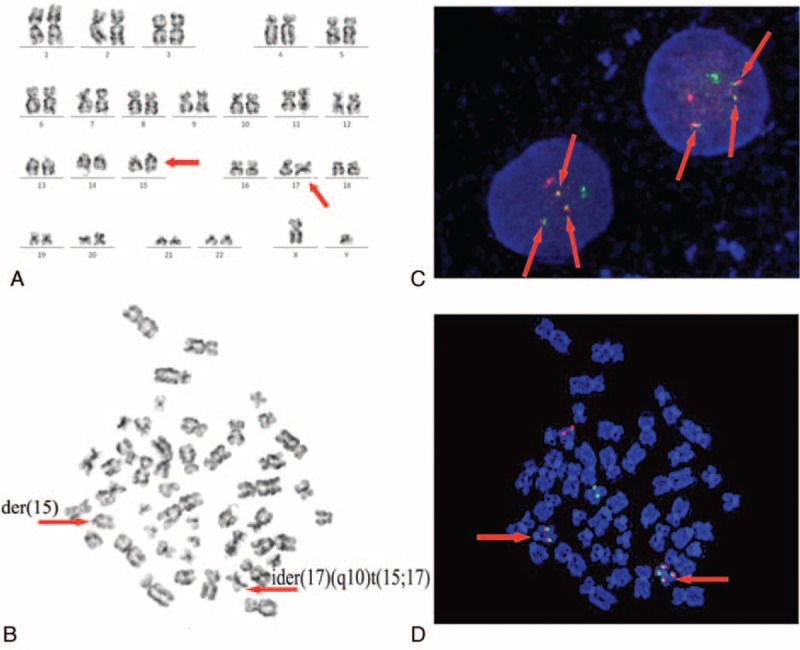
Karyotype and FISH analysis at initial diagnosis. A: the karyotyping from 20 bone marrow metaphase cells showed 46,XY,der(15)t(15;17)(q22;q21),ider (17)(q10)t(15;17). The arrows indicate abnormal chromosomes. B: one of the metaphase karyograms of the bone marrow cells at initial diagnosis. The arrows indicate der(15) and ider(17)(q10)t(15;17). C: the FISH study using an LSI PML-RARA dual-color, dual-fusion translocation probe (Abbott Molecular/Vysis) at diagnosis. The arrows indicate positive PML-RARA fusion signals (3 fusion signals in a cell). D: karyotyping and PML-RARA FISH analysis in the same metaphase cell. FISH = fluorescence in situ hybridization.

A 6-year-old boy was brought to our hospital with the chief complaint of bleeding gums twice and intermittent fever for 3 days in January 2013. He had a fever of 38.2 Celsius, multiple cervical lymphadenopathy, scattered bleeder in both lower limbs and enlarged liver at 2 cm of the subcostal. No special contact, allergies, medicine, or family history were present. No more positive signs were found. The initial blood count showed pancytopenia (Hb, 95 g/L; red blood cell count, 3.18 × 10^12^/L; platelet count, 57 × 10^9^/L; and white blood cell count, 2.23 × 10^9^/L). The bone marrow morphology displayed a hypercellular marrow with increased abnormal promyelocytes, which were variable in the cell size and nucleolus, rich in cytoplasm and varying granules, and visible of round or oval, distorted, folded nucleus, accounting for 87.5% of all nucleated cells. Bone marrow specimens were also positive for myeloperoxidase and nonspecific esterase staining (Fig. 1). Flow cytometry analysis (Fig. 2) with the bone marrow showed that ∼91.5% of blasts were strongly positive for CD9, CD13, CD15, CD33, CD45, CD64, CD123, Myeloperoxidase, with partial expression of CD38, CD117, HLA-DR, CD2, CD3, CD4, CD5, CD7, CD8, CD10, CD11b, CD14, CD16, CD19, CD20, CD22, and CD34, whereas CD56, CD71, GlyA, cCD79a, cCD3, TdT were negative (Tables 1 and 2). Cytogenetic studies showed a karyotype of 46,XY, der(15)t(15;17)(q22;q21), ider(17)(q10)t(15;17) according to ISCN2009^20^ (Fig. 3A and B). Fluorescence in situ hybridization (FISH) with Vysis LSI PML/RARα dual color, dual fusion translocation probe (Abbott Molecular, IL) revealed nuc ish (nuclear in situ hybridization) (PML × 4)(RARA × 4)(PML con RARA × 3)[400] (Fig. 3C and D). The fusion PML-RARA gene in this patient was furthered characterized as bcr3 subtype by real-time quantitative polymerase chain reaction (RT-qPCR). Therefore, the patient was diagnosed as APL according to bone marrow morphology, immunophenotyping, cytogenetics, and molecular biology studies. Subsequently, the patient was administrated with induction chemotherapy consisting of ATRA at a dose of 45 mg/m^2^/d for 33 days, arsenic trioxide 0.15 mg/kg/d for 27 days, and daunorubicin 60 mg/m^2^/d for 3 days.

**TABLE 1:**
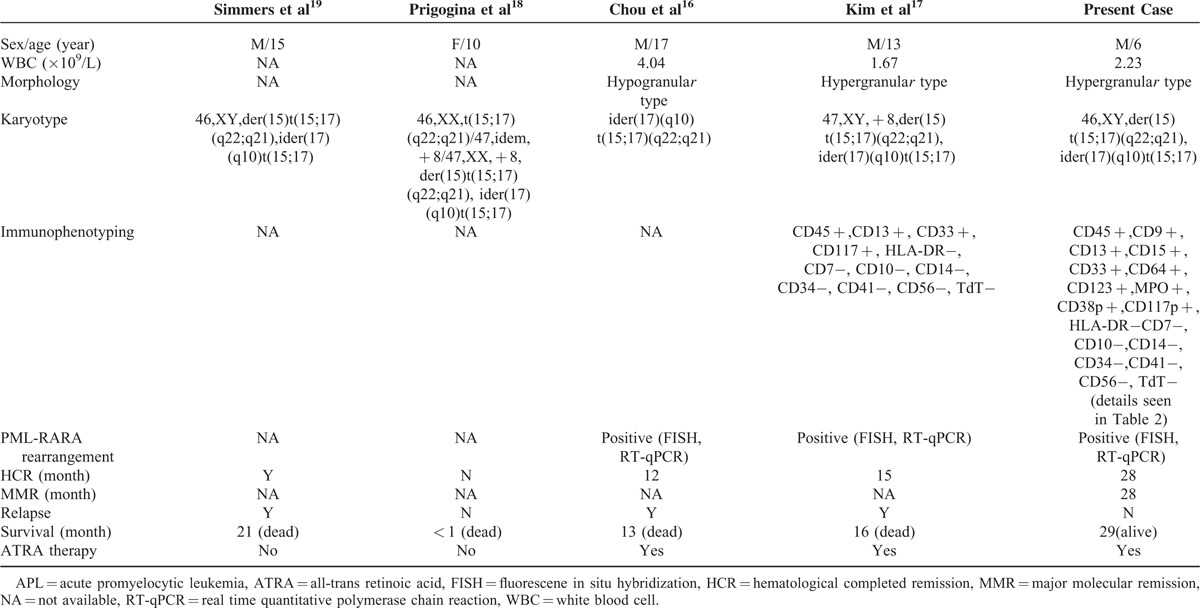
Comparison Between Previous 4 Reports and Present Study With ider(17)(q10)t(15;17)(q22;q12) Positive APL

**TABLE 2:**
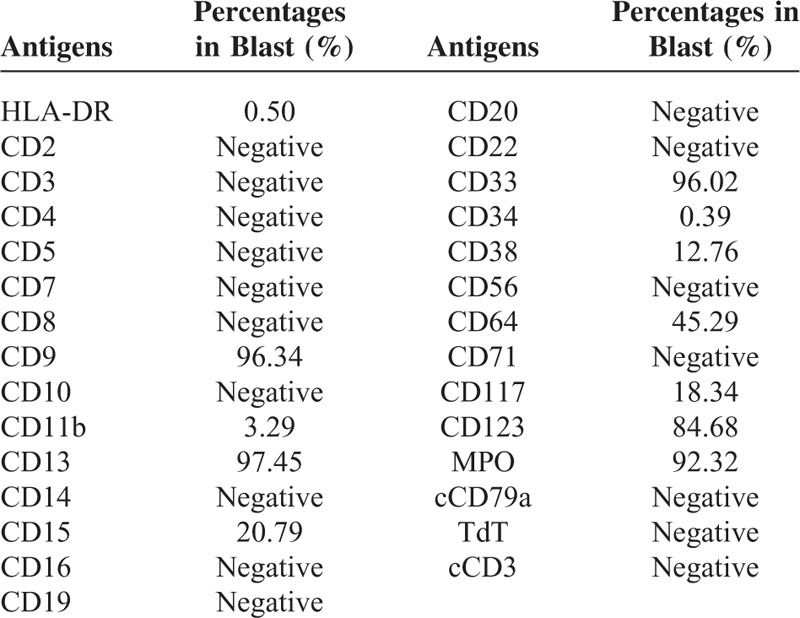
The Percentages of the Detected Antigens in this Patient by Flow Cytometry

After the induction chemotherapy, the blood cell counts came back to normal levels and bone marrow aspirate showed hematological completed remission (HCR) but not major molecular remission (MMR) with detection out of PML-RARA by RT-qPCR. Then, one cycle of DA regimen (Daunorubicin 60 mg/m^2^/d, d1-3;cytarabine 200 mg/m^2^/d, d1-7) and another cycle of DA regimen (Daunorubicin 45 mg/m^2^/d, d1-3;cytarabine 1 g/m^2^/12 h, d1-4) were administrated to the patient as the consolidation chemotherapy. After the consolidation therapy, the patient achieved MMR (the PML-RARA was undetectable by RT-qPCR) under HCR. Maintenance chemotherapy was still continued following the consolidation chemotherapy according to the 2012 NCCN guideline for APL. No serious side reactions occurred in the chemotherapy process. During and after the maintenance chemotherapy, this patient was always under persistent MMR and HCR without any clinical symptoms or signs till now.

The patient provided written informed consent for the publication of this case details and the consent procedure was approved by the ethic committee for Drug Clinical Trial of Huazhong University of Science and Technology.

## DISCUSSION

The ider(17)(q10)t(15;17) is a derived isochromosomal abnormality on the long arm of chromosome 17 with APL unique reciprocal translocation of t(15;17). It is also a rare cytogenetic abnormality in APL. From January 2013 to December 2014, we detected 12 cases with the karyotype of ider(17) (q10)t(15;17) in 635 APL patients with t(15;17) in our center. They are at the average age of 29.6 years (from 6 to 75 years old). The incidence of ider(17) (q10)t(15;17) in our center is 1.89%, which is consistent with previous report.^6^ Although previous studies^11,21^ reported that the ider(17q)(q10)t(15;17) had a slightly higher frequency in APL patients with PML-RARA bcr1 subtype, which might be related to delayed CR and lower sensitivity to ATRA treatment, we found no significant correlation between ider(17) (q10)t(15;17) and 3 isoforms of PML-RARA gene (bcr1, bcr 2 and bcr3 subtypes) in our center, which may be caused by the rarity of ider(17) (q10)t(15;17) since we only have 12 ider(17) (q10)t(15;17) positive cases until now.

To the best of our knowledge, only 4 pediatric APL cases with the ider(17q)(q10)t(15;17) have been reported in the literature so far.^21–23^ The role of the ider(17q)(q10)t(15;17) in APL is still an ongoing investigation and all of the previous studies considered it as a poor prognosis maker for APL especially in pediatric APL patients.^2,6,10,17,21^ As shown in Table 1, no matter the variable clinical and laboratory features as wells as different treatments, 1 of the 4 reported pediatric patients did not achieve CR at all and died in the first month from the initial diagnosis, whereas the remained 3 cases relapsed and died within 13 to 21 survival months.^16–19^ Interestingly, Hu and colleagues^21^ showed that the ider(17)(q10)t(15;17) had a proliferation and growth advantage, which might be mediated by loss of a tumor suppressor TP53 allele. However, no drug-sensitive analysis has been performed in the ider(17)(q10)t(15;17) positive clones and the detailed role of TP53 copy number variation in these clones is still mysterious. The sensitivity of these clones containing the ider(17)(q10)t(15;17) to ATRA, arsentic trioxide and other chemotherapy drugs is still unknown and need further studies.

Interestingly, the case we presented here displayed encouraging clinical outcomes until now. He was treated with both ATRA and arsenic and achieved HCR after induction therapy and MMR after consolidation therapy. After consolidation chemotherapy, this patient was still under continuous HCR and MMR for >28 months until now by detection of MRD every 3 months. Besides, no serious complications and side effects (such as the ATRA syndrome) of chemotherapeutic agents occurred during and after serials of therapies. This is the first case report of pediatric APL with ider(17)(q10)t(15;17) showing favorable outcomes after chemotherapy. It suggests that the ider(17)(q10)t(15;17) may not be the sole determinants for worse outcomes in pediatric APL and more contributed factors need to be considered for pediatric APL prognosis.

As an inference, we think the encouraging clinical outcomes from this case may be attributed to 2 important factors. First, this patient was classified in the low-risk group at the initial diagnosis stage according to the 2012 NCCN guideline for APL. The risk classification is very important for APL, especially pediatric APL, and it correlates very well with the therapeutic effects, clinical outcomes, and relapse. Basically, low-risk classification means better response to treatment and less relapse. Second, we think the patient may also benefit from the early usage of arsenic trioxide. As one of the most famous Chinese traditional medicaments, arsenic trioxide has been shown powerful therapeutic effects in APL and has been the standard treatment for APL patients in both induction and maintenance chemotherapies. Combination of ATRA and arsenic also has synergistic effects in APL patients during induction therapy due to their different pharmacology targets (ATRA targets RARα and arsenic targets PML).^24,25^ For the 4 previously reported pediatric APL cases with the ider(17)(q10)t(15;17), 2 of them^18,19^ were reported in 1986 and 1987, which were before the starting of ATRA treatment in 1988. The remaining 2 cases also did not involve the combination of ATRA and arsenic in the induction chemotherapy.^16,17^ Therefore, we infer that the patient reported here may benefit from the combination of ATRA and arsenic in the induction chemotherapy. However, more case reports and systematic analysis are needed to study the role of ATRA and arsenic in the treatment of pediatric APL patients with the ider(17)(q10)t(15;17).
